# Subcellular trafficking of guanylyl cyclase/natriuretic peptide receptor-A with concurrent generation of intracellular cGMP

**DOI:** 10.1042/BSR20150136

**Published:** 2015-10-30

**Authors:** Indra Mani, Renu Garg, Satyabha Tripathi, Kailash N. Pandey

**Affiliations:** * Department of Physiology, Tulane University Health Sciences Center and School of Medicine, 1430 Tulane Avenue, New Orleans, Louisiana 70112, U.S.A.

**Keywords:** endosomes, guanylyl cyclase/natriuretic peptide receptor-A, immunofluorescence, lysosomes, receptor internalization

## Abstract

Atrial natriuretic peptide (ANP) activates guanylyl cyclase/natriuretic peptide receptor-A (GC-A/NPRA), which lowers blood pressure and blood volume. The objective of the present study was to visualize internalization and trafficking of enhanced GFP (eGFP)-tagged NPRA (eGFP–NPRA) in human embryonic kidney-293 (HEK-293) cells, using immunofluorescence (IF) and co-immunoprecipitation (co-IP) of eGFP–NPRA. Treatment of cells with ANP initiated rapid internalization and co-localization of the receptor with early endosome antigen-1 (EEA-1), which was highest at 5 min and gradually decreased within 30 min. Similarly, co-localization of the receptor was observed with lysosome-associated membrane protein-1 (LAMP-1); however, after treatment with lysosomotropic agents, intracellular accumulation of the receptor gradually increased within 30 min. Co-IP assays confirmed that the localization of internalized receptors occurred with subcellular organelles during the endocytosis of NPRA. Rab 11, which was used as a recycling endosome (Re) marker, indicated that ∼20% of receptors recycled back to the plasma membrane. ANP-treated cells showed a marked increase in the IF of cGMP, whereas receptor was still trafficking into the intracellular compartments. Thus, after ligand binding, NPRA is rapidly internalized and trafficked from the cell surface into endosomes, Res and lysosomes, with concurrent generation of intracellular cGMP.

## Abstract (precis)

Atrial natriuretic peptide (ANP) modulates blood pressure and fluid volume by activation of natriuretic peptide receptor-A (NPRA). Immunofluorescence (IF) studies reveal that NPRA is internalized and redistributed into subcellular compartments with concurrent production of cGMP.

## INTRODUCTION

Atrial natriuretic peptide (ANP) is a cardiac hormone belonging to the natriuretic peptide family, which consists of ANP, brain natriuretic peptide (BNP) and C-type natriuretic peptide (CNP), each derived from a separate gene [1]. ANP exerts various vascular, renal and endocrine effects, resulting in the regulation of blood pressure and extracellular fluid volume homoeostasis [2]. Both ANP and BNP activate guanylyl cyclase/natriuretic peptide receptor-A (GC-A/NPRA), which, in response to hormone binding, produces second-messenger cGMP [3]. CNP activates NPRB, which also produces cGMP. However, all three natriuretic peptides arbitrarily bind to NPRC, which lacks guanylyl cyclase activity [4–8]. After ligand binding, NPRA dimerizes and the guanylyl cyclase catalytic domain probably becomes activated [9]. Generally, cells respond to their environment through binding of extracellular ligand to plasma membrane receptors, which transduce information to the cell interior and activate intricate signalling networks. The amount and duration of receptor signal transduction is strongly regulated by endocytic trafficking, during which receptors are removed from the cell surface by endocytosis, pass through the endosomal system and are either recycled or delivered to lysosomes, where signalling is down-regulated and the receptors are degraded [10,11]. Although the ligand-mediated endocytosis of various ligand–receptor complexes has been described in many cell types, the mode of signalling mechanisms is not well known [12–15].

Specifically, in terms of NPRA signalling, it has been shown that binding of radiolabelled-ANP activates NPRA and initiates its internalization in the cell interior [16–18]. However, the cellular life cycle of NPRA in the context of internalization, recycling and metabolic processing in the subcellular compartments of intact cells has been controversial. It was previously suggested that among the three defined natriuretic peptide receptors, only NPRC was internalized in a ligand-dependent manner [19,20]. The assumption was perpetuated due to the suggestion that ANP–NPRA complexes rapidly dissociate following ANP binding to NPRA at 37°C. However, those studies did not follow the normal binding experimental protocol, in that the dissociation of ^125^I-ANP was carried out in a medium containing high concentrations of unlabelled ANP [19,20]. It was subsequently reported that the absence of ANP/NPRA internalization in those previous studies may have resulted from slow ligand degradation rate in specific cell types [21]. Several studies by us and others utilizing ^125^I-ANP-binding analyses have demonstrated that bound ANP/NPRA complexes are internalized, processed intracellularly and the degraded products released into culture medium [17,22–27]. Furthermore, NPRB has also been shown to be endocytosed and recycled back to the plasma membrane in response to CNP binding [28]. The present work was undertaken to elucidate unequivocally direct visualization of ligand-dependent endocytosis and intracellular trafficking of NPRA by utilizing confocal IF analyses. NPRA is considered to be the biological active receptor of ANP and BNP because most of the physiological effects of these peptide hormones are activated by the production of second messenger cGMP [6,8,29–32]. It has been reported that cGMP interacts with three types of intracellular effector proteins: cGMP-dependent protein kinases, cGMP-regulated ion channels and cGMP-activated phosphodiesterases [6,33]. It is possible that cGMP-binding proteins transduce the cGMP signal to alter cell function through various mechanisms. One such mechanism may be stimulation of protein phosphorylation; another may be inhibition of this process [34,35]. However, the molecular mechanism of ANP/NPRA/cGMP signalling and the fate of the internalized receptors remains incompletely understood.

Intracellular trafficking, visualization and concurrent signalling of NPRA in subcellular compartments have not been previously demonstrated. We prepared the enhanced GFP (eGFP)-tagged NPRA (eGFP–NPRA) construct to visualize the internalization, intracellular trafficking and signalling of receptor in the subcellular compartments to delineate the molecular mechanism of ANP/NPRA/cGMP signalling. In the present study, we used IF staining (IFS) and co-immunoprecipitation (co-IP) of NPRA with plasma membrane, endosomal, lysosomal and Rab 11 markers to follow intracellular trafficking and signalling by confocal IF microscopy (CIF) and immunoblotting (IB). These functional compartments represent the physical basis for the assembly and turnover of signalling complexes, which in turn can define specialized endosomal–lysosomal signalling platforms. The present study provides direct evidence of the internalization and trafficking of NPRA from the cell surface to the intracellular compartments to extend cGMP signalling in a time and space to compensate for rapid receptor recycling back to the plasma membrane and/or lysosomal sorting of receptor in intact human embryonic kidney-293 (HEK-293) cells.

## EXPERIMENTAL

### Materials

ANP (rat-28) was purchased from Bachem Americas. ^125^I-ANP was purchased from PerkinElmer NEN. A QuickChange II site-directed mutagenesis kit was purchased from Stratagene. The HEK-293 cell line was from the A.T.C.C. Tissue culture supplies and 10% goat normal serum were purchased from Invitrogen/Life Technologies. BSA was obtained from Polysciences. Mouse monoclonal eGFP antibody was purchased from Clontech. Rabbit polyclonal antibody early endosome antigen-1 (EEA-1), lysosome associated membrane protein-1 (LAMP-1), protein A/G PLUS-Agarose marker, Texas Red anti-chicken IgY, Texas Red anti-mouse IgG1 and mouse monoclonal antibody β-actin were purchased from Santa Cruz Biotechnology. Plasma membrane marker anti-pan-cadherin and recycling endosome (Re) marker anti-Rab 11 antibodies were obtained from Abcam. DyLight™405 anti-rabbit IgG (H + L) antibody was obtained from Jackson Immuno Research Laboratories. Rabbit polyclonal cGMP antibody was purchased from Antibodies Online and a cGMP complete-ELISA kit was obtained from Enzo Life Sciences. Texas Red anti-rabbit IgG (H + L) and DAPI were obtained from Vector Laboratories. Synthetic oligonucleotides primers were obtained from MWG Operon (Huntsville, AL). pEGFP-N1 vector was purchased from Clontech. Paraformaldehyde, saponin, chloroquine, ammonium chloride, cycloheximide and all other chemicals were reagent grade and obtained from Sigma.

### Methods

#### Construction of eGFP–NPRA chimeric vector

Fusing the C-terminus of murine NPRA cDNA [3] to the N-terminus of eGFP was done by sub-cloning it into p^cGFP-NT^ vector (Clontech) for its expression as an eGFP–NPRA fusion protein. We used site-directed mutagenesis to create a SmaI site to remove the stop codon in NPRA cDNA and bring it into the same reading frame as eGFP, using a Quick Change™ site-directed mutagenesis kit (Stratagene) according to our established methods [36]. Mutagenesis was carried out using sense 5′-GCAGCTCTCGAGCCCGGGCTACTGCCCTGCTATTCC-3′ and antisense 5′-GGAATAGCAGGGCAGTAGCCCGGGCTCGAGAGCTGC-3′ primers. The mutated NPRA fragment was recloned into the mammalian expression vector p^eGFP-NT^. Using Lipofectamine, the neomycin-resistant vector containing eGFP–NPRA chimeric construct was stably transfected in HEK-293 cells [18]. Single cell clones expressing eGFP–NPRA fusion protein were serially selected by limited dilution in medium containing 500 μg/ml neomycin, as previously described [18]. Antibiotic-resistant clones were isolated and screened by flow cytometry and fluorescence microscopy to obtain a single population of HEK-293 clonal cells expressing eGFP–NPRA fusion protein.

#### Production of polyclonal chicken antibody of NPRA

The peptide ETKAVLEEFDGFE, corresponding to 13 C-terminus residues (1015–1027) in the intracellular region of NPRA, was conjugated to keyhole limpet haemocyanin (KHL). The KHL-peptide conjugate (250 μg) was injected into chickens intraperitoneally (i.p.) in the presence of complete Freund's adjuant (GenWay Biotech). After 21 days, chickens were boosted with 100–150 μg of conjugated antigen and incomplete Freund's adjuvant. Thirty and 60 days thereafter, additional boosts were given (total, three boosts). Eggs were harvested and total IgY isolated from the yolks. The total IgY antibody was evaluated for titre and IgY was affinity-purified using the antigen.

#### Stable transfection of eGFP–NPRA construct and ANP treatment

HEK-293 cells were grown in six-well culture plates containing Dulbecco's modified Eagle's medium (DMEM) supplemented with 10% (v/v) FBS. Initially, sub-confluent cells were transfected with eGFP–NPRA cDNAs using Lipofectamine™ reagents. To establish the stably expressing receptor cell lines, 400 μg/ml of geneticin was added to the culture medium after transfection. Antibiotic-resistant clones were isolated and established for receptor expression, using ANP as a ligand as previously reported [18]. For ANP treatment, cells were pre-incubated with 0.2 mM 3-isobutyl-1-methylxanthin (IBMX) at 37°C for 30 min, then treated by the presence or absence of ANP for different periods (1, 5, 10, 15 and 30 min).

#### Cell surface ^125^I-ANP binding assay

HEK-293 cells stably expressing eGFP–NPRA and NPRA, were grown in 6-cm^2^ culture dishes. Confluent cells were washed with assay medium (DMEM containing 0.1% BSA) and labelled with ^125^I-ANP in the absence or presence of a 100-fold excess of unlabelled ANP. After completion of binding at 4°C, free ligand was removed from the dishes by four washes with ice-cold assay medium (2 ml per wash). To determine cell-surface-associated radioactivity, the acid wash procedure was used [36]. After binding was completed, each culture dish received 1 ml of ice-cold acetate buffer (pH 3.5) and cells were kept at 4°C for 2 min. The acid eluates from the dishes were collected and each dish received another 1 ml of ice-cold acid buffer to wash the cells. Both wash solutions were combined to determine acid-sensitive radioactivity. Cells were then dissolved in 1 N NaOH and acid-resistant radioactivity was determined. Acid-sensitive radioactivity was used as an index of cell surface-bound ^125^I-ANP; acid-resistant radioactivity was used as a measure of internalized ligand–receptor complexes.

#### Internalization of ligand-receptor complexes

HEK-293 cells stably expressing eGFP–NPRA and NPRA, were allowed to bind ^125^I-ANP by incubation at 4°C for 60 min. The unbound ^125^I-ANP was removed by washing cells with ice-cold assay medium (four washes with 2 ml per wash). The total cell-associated radioactivity was determined by dissolving cells in 1 N NaOH and counting the radioactivity in the cell lysate. This represented the initial zero time control value of 100%. To permit the internalization of ligand-receptor complexes, cells were quickly warmed to 37°C. At different time intervals, the culture dishes were removed from 37°C and placed on ice and media were collected. The cell-surface-associated radioactivity was removed by washing the cells with ice-cold acetate buffer (pH 3.5) at 4°C. After acid wash, the internalized ^125^I-ANP radioactivity was determined by dissolving cells in 1 N NaOH.

To determine the rate of lysosomal degradation of ligand–receptor complexes, cells were pre-treated with chloroquine (200 μM) at 37°C for 1 h. Cells were allowed to bind ^125^I-ANP at 4°C for 60 min, then washed with assay medium and re-incubated in fresh assay medium at 37°C. The chloroquine treatment was maintained throughout the entire binding and internalization period of the experiment. It should be noted that chloroquine did not alter the binding capacity of ligand to intact HEK-293 cells at 4°C. To assess the internalization of ligand–receptor complexes at the different time intervals, culture dishes were removed from 37°C, the medium collected, surface-associated radioactivity removed by acetate buffer (pH 3.5) and cells dissolved in 1 N NaOH. The radioactivity in acid eluates, cell lysates and culture mediums were considered respectively, to be cell-surface-associated, internalized and released into medium. The quantification of intact and degraded ligand released into the culture medium after internalization of ligand–receptor complexes was done by precipitation of medium with 10% trichloroacetic acid containing 200 μg/ml BSA as carrier. We considered the recovered ^125^I-ANP in trichloroacetic acid precipitate to be intact ^125^I-ANP molecules and those in the supernatant to be degraded [37].

#### Cell permeabilization and immunofluorescence staining

For all IF studies, cells were seeded on cover glasses and grown for 2 days. Cells were treated with ANP, fixed in 4% paraformaldehyde for 30 min, permeabilized in PBS containing 0.1% BSA and 0.2% saponin, then incubated for 10 min at room temperature. Cells were blocked with 1% normal goat serum, 0.1% saponin and 1% BSA in PBS for 1 h at room temperature, then labelled with anti-NPRA (1:1,000), anti-EEA-1 (1:400), anti-LAMP-1 (1:400), or anti-Rab 11 antibodies (1:500) in blocking buffer overnight at 4°C. Cells were incubated with secondary antibody anti-chicken IgY (1:2000) or anti-rabbit IgG (1:5000) conjugated with Texas Red for 2 h at room temperature in the dark and then washed in PBS three times for 15 min each. Cover glasses were allowed to dry and mounted on glass slides with DAPI (Vector Laboratories), then sealed with Fixogum rubber cement. The details of antibodies used in the IFS are listed in Supplementary Table S1.

#### Confocal microscopy

Cells were examined and images acquired using a TCS SP2 confocal laser scanning microscope (Leica Microsystems). In all experiments, images of cells (1024×1024 pixels) were visualized using the same confocal microscope settings (i.e. sequential scans with wavelengths set as follows: blue, 358–461; green, 488–510; red, 594–615), using a 63× Apo-oil immersion objective (NA=1.4) and 60-μm aperture, using the LEICA Scan TCS-SP2 software (Leica Microsystems). The pinhole was adjusted to keep the same size of z-optical sections (1-μm *z*-axis) for all channels. In all experiments, images of cells were acquired as single mid-cellular optical sections and averaged over eight scans/frame.

#### Quantification of NPRA co-localization

MetaMorph co-localization application (Molecular Devices) plugins were used to quantify co-localization between eGFP–NPRA (green channels) and EEA-1, LAMP-1 or Rab 11(red channels) in individual cells. In brief, at least 100 untreated or treated cells (three coverslips per experiment/condition) were scored per condition, using a confocal microscope (Leica) with a 63×/1.4 NA Plan-Apochromat oil immersion objective lens. We used the 488 green channel for eGFP–NPRA and the 594 red channel for Texas Red-conjugated secondary antibody. For quantification of co-localization, a minimum threshold of red and green channels was selected. The background (median) was subtracted from the original images (using process: arithmetic). The actual images were analysed (choose Apps: measure co-localization) and the percentage of co-localization was calculated from the total co-localized green or red area. Graph prism software was used to generate all the bar graphs and statistical analyses of data. The results are presented as the means ± S.E.M. of five independent experiments.

#### Preparation of cell lysates

eGFP–NPRA stably expressing HEK-293 cells were grown in 6 cm^2^ culture dishes and treated for 5, 10, 15 and 30 min with 100 nM ANP. The cell lysate was prepared essentially, as described earlier [18,36]. In brief, cells were washed with 1× PBS solution and resuspended in 400 μl of lysis buffer containing: 25 mM HEPES (pH 7.5), 0.05% 2-mercaptoethanol, 1% Triton X-100, 1 mM sodium vanadate, 10 mM NaF, 1 mM PMSF, 10 μg/ml aprotinin and 10 μg/ml leupeptin. Cell extract was passed through a 1-cc syringe with a 21-gauge needle and centrifuged at 18000 ***g*** for 15 min. The clear cell lysate was collected and stored at–80°C until use [38,39]. The protein concentrations of the lysate were estimated using a Bradford protein detection kit (Bio-Rad).

#### Subcellular fractionation

HEK-293 cells were lysed in a buffer containing 5 volumes of 10 mM sodium phosphate (pH 7.4), 250 mM sucrose, 150 mM NaCl, 5 mM EDTA, 1 mM PMSF, 5 mM benzamidine, 10 μg/ml leupeptin and 10 μg/ml aprotinin, using minor modification of the procedure previously described [40,41]. Briefly, cells were homogenized in the Dounce homogenizer and cellular debris was cleared by centrifugation at 1000 ***g*** for 5 min at 4°C. The supernatant was collected and the pellet suspended in lysis buffer, homogenized and centrifuged. Both supernatants were pooled and centrifuged at 100000 ***g*** for 1 h at 4° C. The supernatant, which represents the cytosolic fraction, was collected. The 100000 ***g*** pellet was washed twice with lysis buffer and resuspended in 1 ml of solubilization buffer containing 0.5% *n*-dodecyl β-D-maltoside (DDM), 75 mM Tris/HCl (pH 8.0), 2 mM EDTA, 5 mM MgCl_2_, 1 mM PMSF, 5 mM benzamidine, 10 μg/ml leupeptin and 10 μg/ml aprotinin, then incubated overnight at 4°C on a rocker. The lysate was centrifuged at 60000 ***g*** for 30 min to separate insoluble fractions from solubilized membranes. Proteins were quantified using the Bradford assay (Bio-Rad) and subjected to IP.

#### Co-immunoprecipitation of NPRA

For co-IP of NPRA with plasma membranes, early endosomes, lysosomes and Res, cells were fractionated as described, by subcellular fractionation. Protein from solubilized membrane and the cytosolic fraction resulting from 100000 ***g*** centrifugation was quantified (Bio-Rad); 50 μg of protein samples were used for IB analysis representing the input before IP. In all cases, 500 *μ*g of solubilized membranes or cytosolic fractions were incubated with 4 μg of the primary antibodies for 4 h at 4°C on a rocker, after which, 50 μl of agarose conjugate suspension (protein A/G agarose) was added. Samples were incubated overnight at 4°C on a rocker platform for 18 h. The supernatant was removed by centrifugation at 3600 ***g*** for 1 min at 4°C. The beads were washed three times with a buffer containing 1 mM Tris/HCl (pH 7.5), 1 mM EDTA, 150 mM NaCl, 0.1% Triton X-100 and 10% glycine; after each wash, they were centrifuged at 3000 rpm for 1 min at 4°C. The pellet was resuspended in 50 μl of 2× electrophoresis sample buffer, boiled for 5 min and subjected to SDS/PAGE. The details of antibodies used in IP assay are listed in Supplementary Table S1.

#### Western blot analysis

Cells were treated for 5, 10, 15 and 30 min with 100 nM ANP in the presence of 0.2 mM IBMX. Cell lysates were prepared as described previously [38,39]. For electrophoresis, cell lysate (50 μg of protein) was mixed with sample loading buffer, boiled and resolved by SDS/PAGE (10% gel). Proteins were electrophoretically transferred on to a PVDF membrane, which was then blocked with 5% fat-free milk solution in 1× Tris-buffered saline–Tween 20 (TBST) for 2 h at room temperature. The membrane was incubated with primary antibody of NPRA (1:1000), eGFP (1:500), pan-cadherin (1:500), EEA-1 (1:1000), LAMP-1 (1:500) and Rab 11(1:1000) overnight at 4°C in blocking solution and treated with secondary horseradish peroxidase (HRP)-conjugated antibody (1:5000) for 2 h at room temperature. Protein bands were visualized using ECL plus a detection system from Alpha-Innotech. The density of protein bands was determined using the Alpha Innotech Imaging System. The details of antibodies used in the Western blot assay are listed in Supplementary Table S1.

#### Co-immunofluorescence of cGMP with EEA-1

To visualize intracellular accumulations of cGMP, IFS was done as described previously [42,43], with minor modification. Cells were treated with 100 nM ANP for1, 5, 10, 15 or 30 min in the presence of 0.2 mM IBMX and then fixed in 4% paraformaldehyde for 30 min, permeabilized in PBS containing 0.1% BSA/0.2% saponin and incubated for 10 min at room temperature. Cells were blocked with 1% normal goat serum, 0.1% saponin and 1% BSA in PBS for 1 h at room temperature, then labelled with anti-cGMP antibodies (1:1000) and anti-EEA-1 (1:200) in blocking buffer overnight at 4°C. Samples were incubated with secondary antibody anti-rabbit IgG (1:200) conjugated with DyLight™405 and anti-mouse IgG_1_ (1:4000) conjuga-ted with Texas Red for 2 h at room temperature in the dark. The cells were washed three times for 15 min each in PBS. Cover glasses were allowed to dry and mounted on glass slides with Vectashield mounting media (Vector Laboratories), then sealed with Fixogum rubber cement.

#### cGMP assay

Cells were treated with 100 nM ANP for 5, 10, 15 or 30 min in the presence of 0.2 mM IBMX. Cells were washed three times with PBS and scraped in 0.1 M HCl. Cell suspensions were subjected to five cycles of freeze and thaw and then centrifuged at 10000 ***g*** for 15 min at 4°C. The supernatant was collected for cGMP assay using direct cGMP complete-EIA kit (Enzo Life Sciences) according to the manufacturer's protocol.

#### Statistical analysis

Results are presented as mean ± S.E.M. of the average responses in multiple experiments done with different cell preparations. Results were normalized relative to untreated controls. Statistical significance was assessed using ANOVA, followed by Dunnett's multiple comparisons test. The probability value of *P*<0.05 was considered significant.

## RESULTS

### Expression and visualization of ANP-induced internalization of eGFP–NPRA and NPRA

The expression level of chimeric receptor protein eGFP–NPRA was analysed by Western blot using eGFP antibody. The whole-cell extract prepared from cells expressing eGFP–NPRA fusion construct showed the 162-kDa chimeric protein of predicted size. The extract from cells expressing eGFP construct alone, which was used as a positive control, showed expression of 27-kDa eGFP (Figure 1A). The extract from non-transfected cells was used as a negative control.

**Figure 1:**
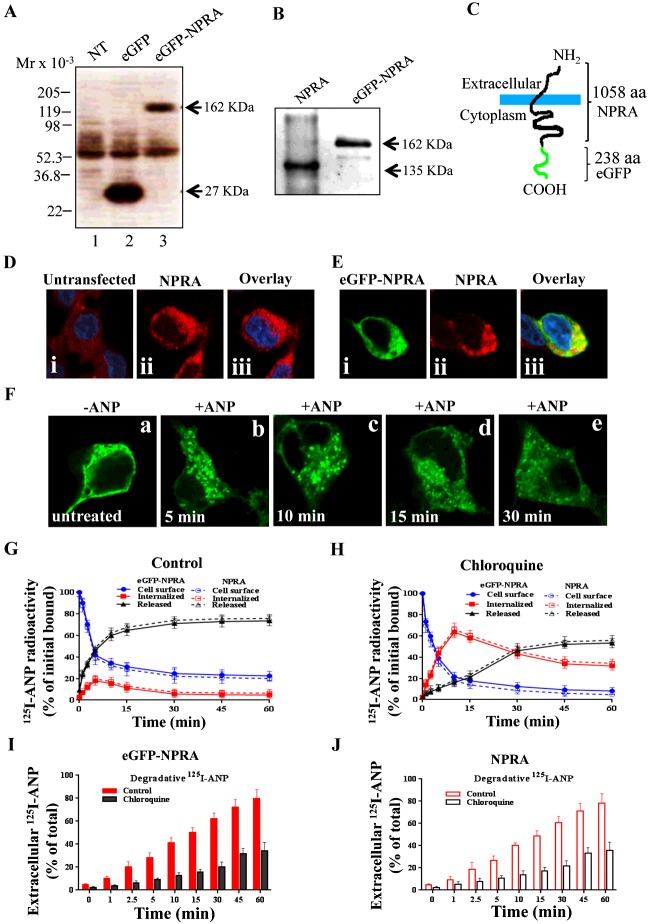
Identification and localization of eGFP–NPRA fusion protein and kinetics of ANP-induced eGFP–NPRA and NPRA internalization in stably expressing recombinant HEK-293 cells (**A**) Western blot analysis indicating the 162-kDa eGFP–NPRA fusion protein. (**B**) Western blot analyses of eGFP–NPRA and NPRA in stably expressing HEK-293 cells detected by using anti-NPRA antibody. (**C**) Schematic representation of eGFP–NPRA fusion protein. (**D**) The localization of NPRA through anti-NPRA antibody staining (Texas Red-conjugated secondary antibodies); (i) NPRA untransfected cells (overlay of anti-NPRA antibody staining and DAPI (blue) (ii) NPRA transfected cells (iii) overlay of anti-NPRA antibody staining and DAPI. (**E**) (i) localization of eGFP–NPRA (green fluorescence) (ii) localization of NPRA through anti-NPRA antibody staining; and (iii) overlay of anti-NPRA antibody staining, eGFP–NPRA and DAPI. (**F**) Series of single confocal plane images were taken from cells fixed with 4.0% formaldehyde to visualize the internalization of NPRA after stimulation by 100 nM ANP. These images of mid-focal planes are from five independent experiments. For the kinetic analyses of receptors, eGFP–NPRA and NPRA stably expressing cells, were pre-treated in the absence or presence of 200 μM chloroquine at 37°C for 1 h. Both control (**G**) and chloroquine-pre-treated (**H**) cells were allowed to bind ^125^I-ANP at 4°C for 60 min. Cells were washed four times with ice-cold assay medium (2 ml per wash) to remove unbound ligand, then warmed to 37°C. At the indicated time intervals, cell-surface-associated (●, ○), internalized (■, □) and released (▲, ∆) ^125^I-ANP radioactivity levels were determined in acid eluates, cell extracts and culture medium. (**I** and **J**) The composition of degraded ^125^I-ANP in culture medium was analysed by determining the trichloroacetic acid-soluble degraded ^125^I-ANP in supernatant. Each data point represents the mean ± S.E.M. of six separate experiments in triplicate dishes.

Expression of eGFP–NPRA fusion protein and NPRA in stably expressing HEK-293 cells was detected using the anti-NPRA antibody through a Western blot (Figure 1B). A schematic representation of the eGFP–NPRA fusion protein is shown in Figure 1(C). Localization of eGFP–NPRA fusion protein and NPRA in stably expressing HEK-293 cells was detected using the anti-NPRA antibody through a confocal microscope (Figures 1D and 1E). To visualize the eGFP-tagged receptor, cells were treated with 100 nM ANP for 5, 10, 15 and 30 min (Figure 1F).

### Internalization and sequestration of ligand-receptor complexes of ANP–NPRA in HEK-293 cells

After binding of ^125^I-ANP to eGFP–NPRA and NPRA, the ligand–receptor complexes were internalized and degraded ligands were released into culture medium. Chloroquine (200 μM), a lysosomotropic agent, significantly inhibited the intracellular degradation of internalized ^125^I-ANP. For these experiments, cells were pre-treated with chloroquine at 37°C, cooled to 4°C and their surface receptors labelled with ^125^I-ANP for 1 h. After the removal of unbound ligand, cells were rapidly warmed to 37°C in fresh medium. At the indicated time points (1, 2.5, 5, 10, 15, 30, 45 and 60 min), the levels of radioactivity associated with the cell surface, internalized into the cell interior and released into the culture medium were quantified using the acid-wash procedure, which specifically dissociated cell surface-bound ^125^I-ANP. The intracellular (acid-resistant) ^125^I-ANP radioactivity increased rapidly to 20% in 5 min. However, after 10 min of incubation of the cells at 37°C, acid-resistant radioactivity decreased to 5% in 30 min and then remained, at a steady state, up to 60 min. The release of radioactivity into the culture medium increased gradually, reaching equilibrium in 30–60 min (Figure 1G). The intracellular (acid-resistant) ^125^I-ANP radioactivity increased to 60% in 10 min in chloroquine-treated cells; in contrast, this radioactivity reached only 20% in control groups. However, after a 15-min incubation of cells at 37°C, the effect of chloroquine was reduced and acid-resistant radioactivity decreased to 40% in 45 min it then remained at a steady-state level for 60 min. The release of radioactivity into the culture medium increased progressively, reaching equilibrium in 30–45 min. Initially, chloroquine time-dependently inhibited the release of ^125^I-ANP, but after a longer incubation time the release of radiolabelled ligand increased steadily. Nevertheless, the treatment of cells with chloroquine significantly blocked the degradation of internalized ^125^I-ANP as compared with control cells (Figure 1H).

Degraded ligand released into the culture medium was quantified by measuring the solubility of ^125^I-ANP products in 10% trichloroacetic acid. The supernatants (containing degraded ligand) were separated by centrifugation. A release of ^125^I-ANP radioactivity in the culture medium accounted for approximately 80% of the total radioactivity. In chloroquine-pre-treated cells, the release of ^125^I-ANP radioactivity was minimal during the initial incubation period but, after 15 min, the radioactivity began to appear and steadily increased in the culture medium. After 30 min incubation at 37°C, the ^125^I-ANP radioactivity released into the culture medium by cells consisted of ∼70%–80% degraded products. However, in chloroquine-treated cells, after the same incubation periods, the released ^125^I-ANP radioactivity consisted of 55%–60% degraded products (Figures 1I and 1J). Along with the eGFP–NPRA, we also performed the eGFP-untagged NPRA internalization kinetics and sequestration of ligand–receptor complexes of ANP–NPRA in HEK-293 cells to demonstrate that eGFP moiety did not hindrance the endocytosis process of receptor. Kinetics and sequestration of eGFP–NPRA and NPRA are demonstrated in similar fashion (Figures 1G–1J).

### Internalization and co-localization of eGFP–NPRA with early endosomal marker EEA-1

To examine the co-localization of eGFP–NPRA with early endosome marker EEA-1, cells were treated with 100 nM ANP for 5, 10, 15 and 30 min. The co-localization of eGFP–NPRA was observed with EEA-1 marker in early endosomes. After internalization, receptor was sequestered with early endosomes; sites of co-localization of receptor and EEA-1 were depicted as yellow foci (Figure 2A). The co-localization of eGFP–NPRA with EEA-1, analysed as 63% at 5 min, gradually decreased to 43% at 10 min, 31% at 15 min and 23% at 30 min. Localization of eGFP–NPRA with EEA-1 was greatest at 5 min and almost complete at 10 min, then gradually decreased from 15 to 30 min (Figure 2B).

**Figure 2:**
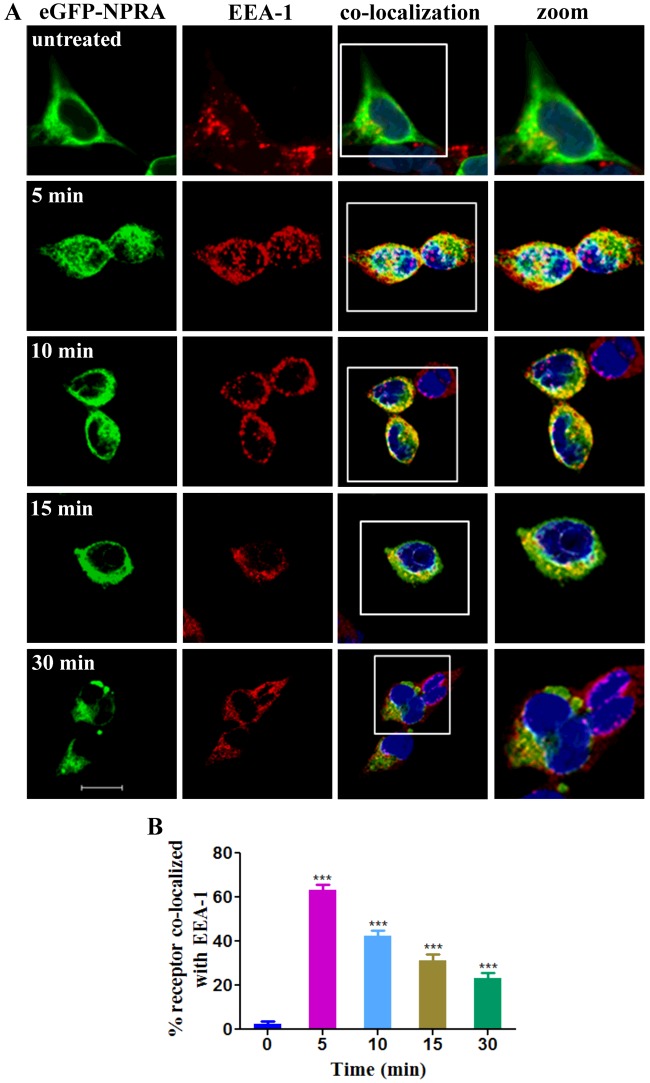
Internalization and co-localization of eGFP–NPRA with EEA-1 in HEK-293 cells (**A**) In untreated cells, all receptors were localized in the plasma membrane. Cells were treated with 100 nM ANP for 5, 10, 15 or 30 min. Co-localization of eGFP–NPRA and EEA-1 marker was observed after 5 and 10 min of treatment with ANP, after which it gradually decreased from 15 to 30 min. The images represent mid-focal planes and five independent experiments. (**B**) Quantification of the percent of co-localization of eGFP–NPRA with EEA-1. Bars represent the mean ± S.E.M. ***P<0.001 relative to untreated cells. Scale bar=50 μm.

### Co-localization of eGFP–NPRA with lysosomal marker LAMP-1 in the presence or absence of lysosomotropic agents chloroquine and ammonium chloride

The internalization route was tracked by co-localization of eGFP–NPRA with the known lysosomal marker LAMP-1, which identifies lysosomes (Figure 3A). To determine the co-localization of eGFP–NPRA with LAMP-1 in lysosomes, cells were treated with 100 nM ANP for different periods. After treatment, the co-localization of receptor with LAMP-1 increased at 5 min (10%), 10 min (20%), 15 min (25%) and 30 min (29%). The co-localization measurement value demonstrated that accumulation of eGFP-NPRA with LAMP gradually increased after 10 min and was almost complete at 30 min (Figure 3B). After internalization, a population of receptors was degraded in lysosomes. To confirm that eGFP–NPRA degradation was mainly dependent on the lysosomal pathway; we used chloroquine and ammonium chloride, inhibitors of lysosomal-dependent degradation. Receptor degradation was indeed prevented by chloroquine (200 μM), as shown by the accumulation of eGFP–NPRA in large perinuclear compartments that co-localized with LAMP-1 (Supplementary Figure S1A). After 10 min of treatment with ANP, eGFP–NPRA was completely internalized and contained in round perinuclear subcellular compartments. The receptor was largely co-localized with LAMP-1, suggesting that it had been routed to lysosomes. In chloroquine-pre-treated cells, ANP-dependent receptor co-localization was observed with LAMP-1 at 5 min (20%), 10 min (37%), 15 min (47%) and 30 min (54%). However, after 10 min of ANP treatment, eGFP–NPRA levels were significantly increased in lysosomal compartments due to inhibition of receptor degradation (Supplementary Figure S1B).

**Figure 3:**
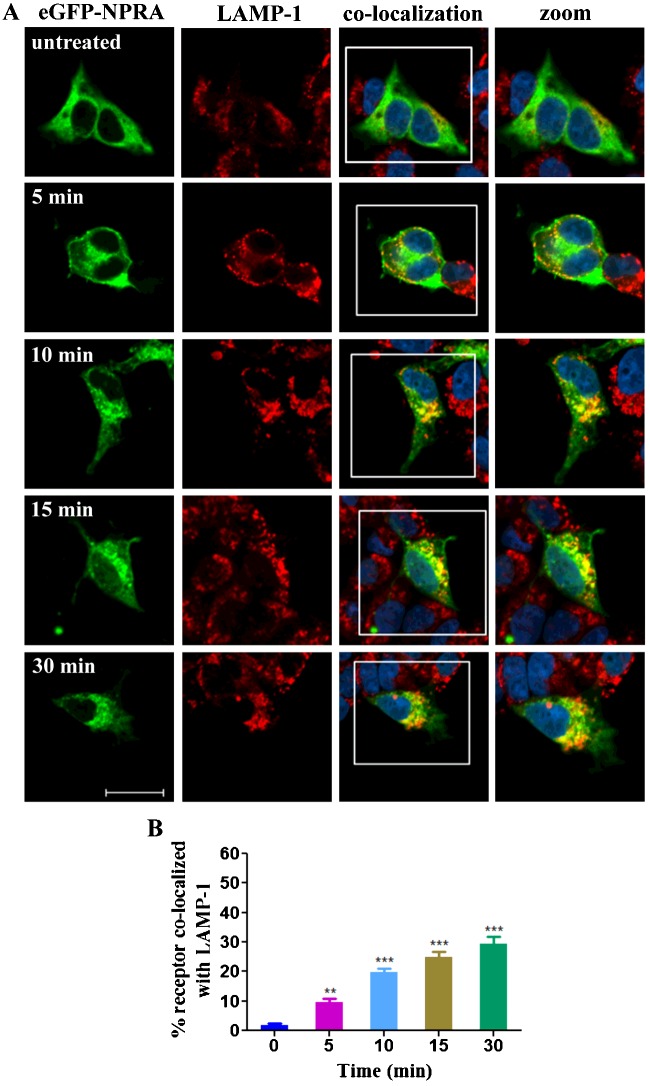
Co-localization of internalized eGFP–NPRA with LAMP-1 in HEK-293 cells (**A**) In untreated cells, all receptors were localized in the plasma membrane. Cells were treated with 100 nM ANP for 5, 10, 15 or 30 min. Co-localization of eGFP–NPRA with LAMP-1 marker gradually increased from 5 to 30 min after treatment. The images represent mid-focal planes and are typical of 4–5 independent experiments. (**B**) Quantification of the percent co-localization of eGFP–NPRA with LAMP-1. Bars represent the mean ± S.E.M. ***P<0.001 relative to untreated cells. Scale bar=50 μm.

The results presented in Supplementary Figure S2(A) show the co-localization of eGFP–NPRA with LAMP-1 after treatment with the lysosomotropic agent ammonium chloride (10 mM). Treatment with ANP for different times accelerated the endocytosis of receptor and most receptors were co-localized with LAMP-1. The receptor density in lysosomes increased at 5 min (18%), 10 min (35%), 15 min (44%) and 30 min (52%) (Supplementary Figure S2B). Treatment of cells with the lysosomotropic agents chloroquine and ammonium chloride significantly blocked the degradation of NPRA as compared with that in untreated control cells and restored the eGFP–NPRA protein levels in lysosomal compartments.

### Co-localization of eGFP–NPRA with recycling endosome marker Rab 11

Co-localization of eGFP–NPRA with Rab 11 showed that NPRA and Rab 11 both reside on shared vesicles (Figure 4A). The percent co-localization value of eGFP–NPRA with Rab 11 showed that receptor recycling occurred at 5 min (13%), 10 min (19%), 15 min (17%) and 30 min (5%; Figure 4B). On the other hand, after internalization, receptor trafficked with early endosomes and reached its maximum at 5 min. Similarly, after endosomal routing, receptor trafficked with lysosomal compartments, as shown by using the lysosomotrophic agents, chloroquine and ammonium chloride. A small population of receptor escaped the lysosomal compartments and, after early endosome routing, recycled back to the plasma membrane through Res (Supplementary Table S2).

**Figure 4:**
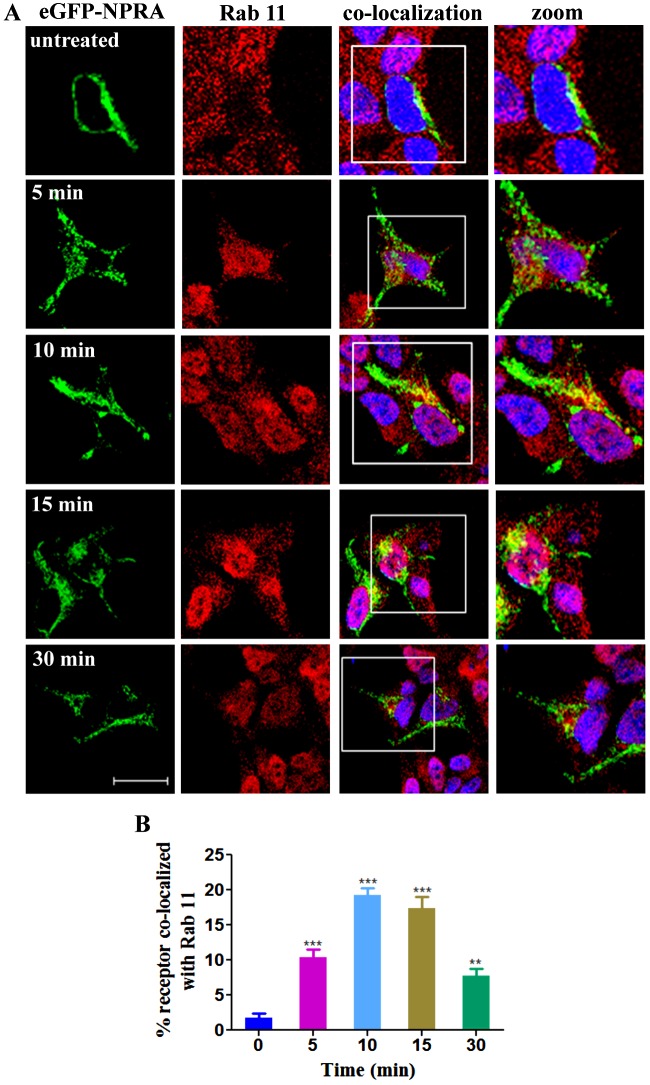
Co-localization of eGFP-NPRA with Rab11 in HEK-293 cells (**A**) In untreated cells, all receptors were localized in the plasma membrane. Cells were treated with 100 nM ANP for 5, 10, 15 or 30 min. Co-localization of eGFP–NPRA and Rab 11 marker was observed after 10 and 30 min of treatment with ANP. Sites of co-localization for receptors and Rab11 are depicted as yellow foci. The images represent mid-focal planes and are typical of four independent experiments. (**B**) Quantification of the percent of co-localization of eGFP–NPRA with Rab11. Bars represent the mean ± S.E.M. ***P<0.001 relative to untreated cells. Scale bar=50 μm.

### Lysosomal degradation of NPRA and intracellular accumulation of cGMP

Western blot analysis showed that receptor degradation occurred in lysosomal compartments after treatment with 100 nM ANP (Figure 5A). However, treatment of cells with the lysosomotropic agents, chloroquine and ammonium chloride, significantly blocked degradation of NPRA and restored the accumulation of eGFP–NPRA fusion protein levels inside treated cells as compared with untreated control cells (Figure 5B). To visualize concurrent generation of cGMP in the intracellular compartments whereas the receptor was trafficking, we did co-IF of cGMP with early endosome marker EEA-1, thus showing that intracellular accumulation of cGMP occurred in the early endosomes (Figure 5C). Intracellular accumulation of cGMP was visualized using fluorescence intensity by measuring the cGMP IF at different time points in intact cells that had been treated with 100 nM ANP at 1, 5, 10, 15 and 30 min. Increased staining was observed inside treated cells, as compared with untreated ones, along with significant enhancement of cGMP fluorescence intensity, at 5 min (19-fold), 10 min (27-fold), 15 min (25-fold) and 30 min (18-fold) (Figure 5D). However, at 30 min, the diffused fluorescence of cGMP was observed in the cytoplasm. ANP time-dependently stimulated the intracellular accumulation of cGMP at 5, 10, 15 and 30 min, with maximum levels occurring at 10 min and then declining until 30 min (Figure 5E).

**Figure 5:**
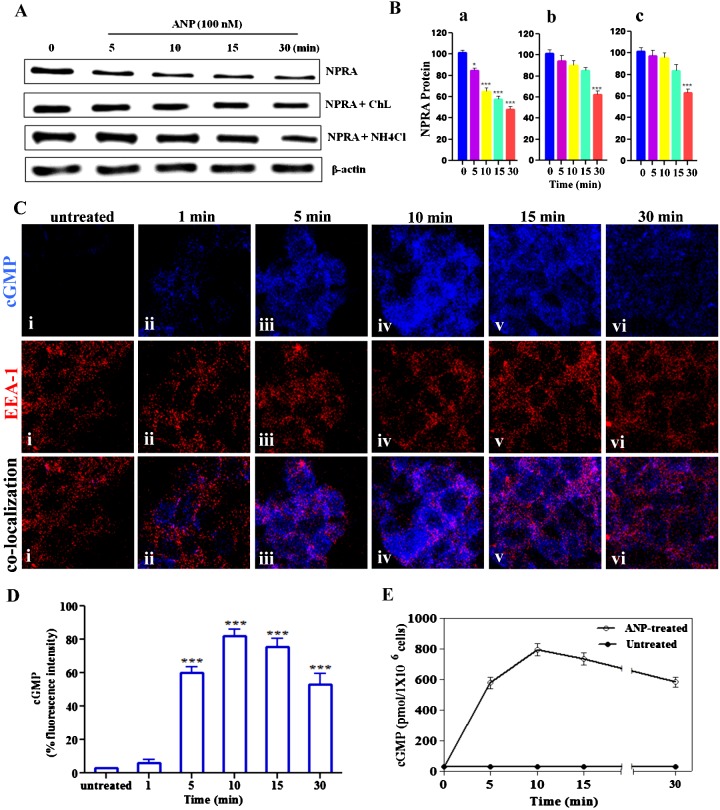
Lysosomal degradation of receptors in the presence or absence of lysosomotropic agents (**A**) Analysis of eGFP–NPRA fusion protein degradation and effect of lysosomotropic agents. Cells were treated with 100 nM ANP for 5, 10, 15 or 30 min. Inhibition of eGFP–NPRA degradation was determined by Western blot after treatment with chloroquine (200 μM) and ammonium chloride (10 mM, both lysosomotropic agents). (**B**) Densitometric western blot quantification of (**a**) NPRA; (**b**) NPRA + ChL; (**c**) NPRA + NH_4_Cl relative to untreated cells. (**C**) Co-IF analysis of the co-localization of cGMP with early endosomes in intact cells. (**i**) Untreated cells stained with DyLight™405 anti-rabbit antibody without prior incubation with the first rabbit antiserum. (**ii**–**vi**) Cells were treated with ANP for 1, 5, 10, 15 or 30 min, which show the co-localization (pink) of cGMP (blue) with EEA-1 (red). Co-localization of cGMP with EEA-1 clearly showed the concurrent generation of signal whereas receptor was trafficking. (**D**) Bars represent the densitometric analysis of the cGMP fluorescence intensities. (**E**) Stimulation of intracellular accumulation of cGMP in cells treated with 100 nM ANP. Cells were treated for 5, 10, 15 or 30 min at 37°C in the presence of IBMX. For each time point, untreated cells were used as controls. cGMP was quantified by ELISA. The images shown are typical of 4–5 independent experiments. Bars represent mean ± S.E.M. ***P<0.001 relative to untreated cells. Scale bar=50 μm.

### Co-immunoprecipitation of NPRA with Pan-cadherin, EEA-1, LAMP-1 and Rab 11

Biochemical assays were used to test the results of IF experiments. Co-IP results clearly indicated that NPRA was co-localized with pan-cadherin, EEA-1, LAMP-1 and Rab 11 compartments during endocytic events, after treatment with 100 nM ANP. Decreased co-localization of receptors with plasma membranes (NPRA-pan-cadherin complex) was examined after increasing the time-course (Figures 6A and 6B). The association of NPRA with early endosomes (NPRA–EEA-1 complex) was greatest at 5 min and then gradually decreased (Figures 6C and 6D). The association of NPRA with lysosomes (NPRA–LAMP-1complex) gradually increased after 5 min, reaching maximum at 30 min (Figures 6E and 6F). The greatest association of receptors with Res (NPRA–Rab 11 complex) occurred at 10 min (Figures 6G and 6H).

**Figure 6:**
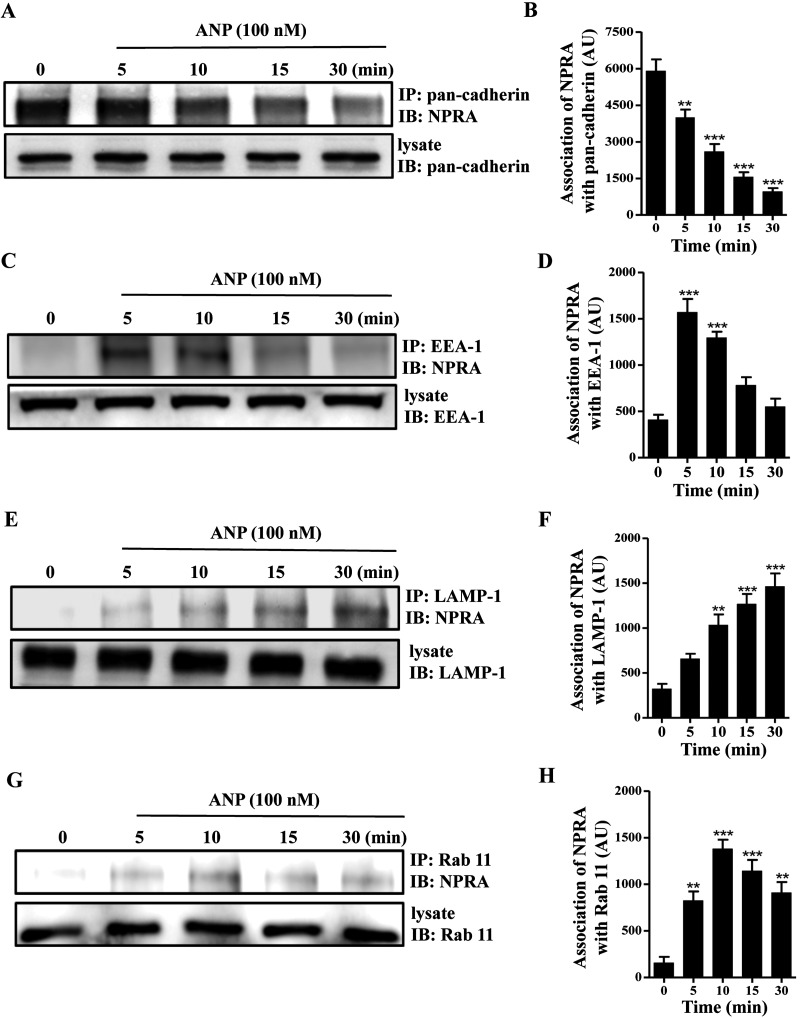
Subcellular fractionation and co-IP of NPRA with pan-cadherin, EEA-1, LAMP-1 and Rab 11 in HEK-293 cells To determine the association of NPRA with pan-cadherin, EEA-1, LAMP-1 and Rab 11, cells were stimulated with 100 nM ANP for different times. To confirm that lysate contained similar amounts of pan-cadherin, early endosome, lysosome and Res, equal amounts of proteins were immunoblotted with antibodies against each of the entities after subcellular fractionations. (**A**) Immunoblot of NPRA after IP of pan-cadherin, showed decreased association with increasing time. (**B**) Densitometric western blot quantification of NPRA association with pan-cadherin relative to untreated cells. (**C**) Co-IP of NPRA with EEA-1 showed maximum association at 5 min, after which it gradually decreased. (**D**) Densitometric western blot quantification of NPRA with EEA-1 relative to untreated cells. (**E**) Association of NPRA with lysosome gradually increased after 5 min, reaching its maximum at 30 min. (**F**) Densitometric western blot quantification of NPRA with LAMP-1 relative to untreated cells. (**G**) Strong association of receptor and Res was observed at 10 min, after which it gradually decreased. (**H**) Densitometric western blot quantification of NPRA with Rab 11 relative to untreated cells. The quantification of western blot results are presented in arbitrary units (AU). Bars represent the mean ± S.E.M. of five independent experiments.*P<0.05; **P<0.01; ***P<0.001 relative to untreated cells.

## DISCUSSION

Our present findings demonstrate that the eGFP–NPRA fusion protein of the receptor was internalized and trafficked with early endosomes. The co-localization of receptor with EEA-1 was at its maximum at 5 min and still almost complete at 10 min, then gradually decreased up to 30 min. Endosomal processing of macromolecules internalized by receptor-mediated endocytosis has been reported in different cell types [44–46]. Selective removal of receptor is mediated by internalization from the plasma membrane, followed by sorting into intraluminal vesicles of late endosomes or multivesicular bodies (MVBs) and subsequent delivery to the lysosomal lumen for degradation [10,47–50]. The proposed schematic representation demonstrates the itinerary of NPRA internalization, intracellular trafficking, recycling and degradation of ligand receptor complexes from the cell surface to cell interior and back to the plasma membrane (Figure 7). Our results demonstrate that after trafficking with endosomes, eGFP–NPRA routed with lysosome. The accumulation of eGFP–NPRA in intracellular compartments was observed after treatment of cells with the lysosomotropic agents chloroquine and ammonium chloride. In ANP-treated cells, eGFP–NPRA was time-dependently co-localized with LAMP-1. Chloroquine and ammonium chloride inhibit lysosomal degradation of intracellular trafficking of the ligand–receptor complexes [37,51]. However, the lysosomotropic agents did not completely block receptor degradation in lysosomes, suggesting that the trafficking of intact eGFP–NPRA also occurred through a lysosome-independent pathway.

**Figure 7:**
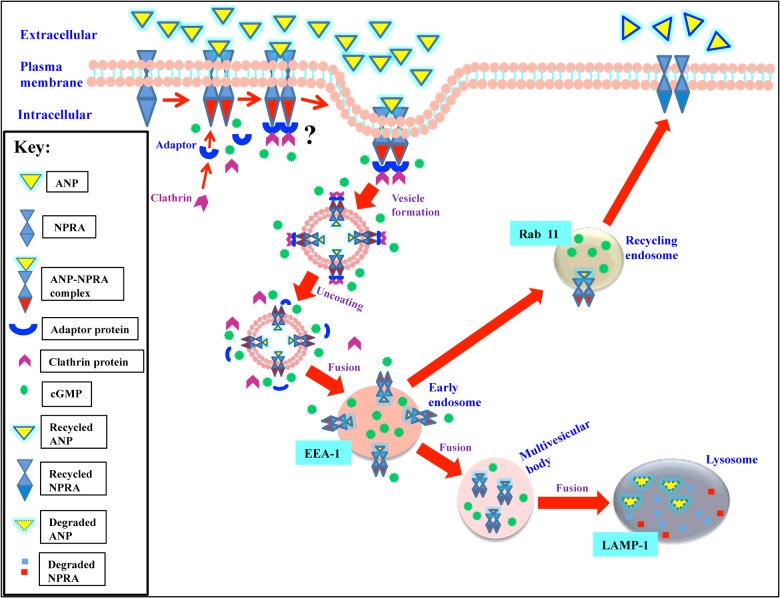
Schematic representation of intracellular trafficking of NPRA in HEK-293 cells The scheme depicts the pathways of internalization, trafficking, recycling and degradation of ligand–receptor complexes. After binding of ligand (yellow), the receptor (blue) is activated and intracellular cGMP (green) is produced. The ligand–receptor complex enters the cell via coated pits. The ligand-bound receptor complex is trafficked intracellularly through endosomes and lysosomes. A small population of receptor recycles back to the plasma membrane through Res along with the concurrent generation of intracellular cGMP. Sorting of bound ANP–NPRA complexes (shown in a disrupted triangle in yellow for ligand and rectangles of blue and red colour for NPRA) occurs by endosomal-dissociation metabolic and lysosomal degradative pathways. Note that the multi-vesicular body formation probably places the receptor in the lumen of the lysosomes.

To examine the recycling of internalized receptor back to the plasma membrane, we used Rab 11 as a marker for slow-Res. IF and Co-IP data clearly demonstrated that ∼20% of receptor recycled back to the plasma membrane. In IF, Rab11 (a member of the Ras superfamily) was mainly detected in the perinuclear region, but a very small amount of receptor co-localization was observed with Rab11. Specifically, Rab11 is one of the extensively studied Rab GTPases; it primarily associates with Res and regulates the recycling of endocytosed proteins [52–55]. Our findings demonstrate the association of Rab 11 with NPRA, indicating a novel mechanistic process of NPRA trafficking into the subcellular compartments. The recycling of internalized receptor back to the plasma membrane occurs simultaneously with this process, leading to degradation of the majority of ligand–receptor complexes into lysosomes.

Confocal IF of ligand-induced receptor co-localization with subcellular organelles was observed as yellow fluorescence, strongly supporting the notion that the association of receptor protein occurs with the subcellular compartments. The co-IP studies also showed that after internalization, the receptor shared subcellular compartments during intracellular trafficking. These results provide strong evidence of the association of NPRA with subcellular organelles. The association of other receptors with subcellular organelles has also been reported, demonstrating the interaction of p97 ATPase with EEA1 in early endosomes [56], the association of arrestin with G-protein-coupled receptor (GPCR) in endo-lysosomal compartments [57] and co-IP of human angiotensin II type I receptor with Rab 11 in Res [58].

The fusion protein eGFP–NPRA generated optimum levels of intracellular accumulation of cGMP in intact cells, indicating that the eGFP moiety did not sterically hinder the biological activity of NPRA. The expression of fully functional eGFP–NPRA permitted detailed analyses of the consequences of receptor internalization and trafficking in HEK-293 cells. The present results demonstrate that the internalization of eGFP–NPRA was time-dependently triggered by ANP treatment; however, in control cells without ANP treatment, receptors were localized on the plasma membrane. Our data show that ^125^I-ANP binds to cell-surface eGFP–NPRA and NPRA, enters through the process of receptor-mediated endocytosis and is delivered to the intracellular compartments until, after 30 min, it reaches a steady-state level. The distribution of ^125^I-ANP radioactivity on the cell surface, in intracellular compartments and in culture medium showed a dynamic equilibrium among the rates of ^125^I-ANP uptake, subcellular sequestration, degradation and extrusion from the cell interior to extracellular spaces. The ^125^I-ANP-binding assay was used to support the internalization kinetics and cellular trafficking of the eGFP–NPRA fusion protein in intact cells. Together, the results of IF analyses and ^125^I-ANP-binding assays established that the homoeostatic regulation of native NPRA and cellular sensitivity of ANP depend on a dynamic equilibrium and reuse of ligand–receptor complexes from the cell surface to the cell interior.

Our results show that ligand–receptor complexes continue to signal by producing cGMP even after receptor mobilization into endosomes. Moreover, cGMP regulates several processes, including cellular growth and contractility, cardiovascular homoeostasis, inflammation, sensory transduction, neuronal plasticity and learning [59]. Intriguingly, we found that cGMP production is not activated exclusively at the cell surface, but also occurs even when receptors have been internalized and continue trafficking in the subcellular compartments. Earlier reports have indicated that GPCRs continue signalling by generation of cAMP during and after internalization processes with their ligands [60]. Signalling from inside the cell is persistent, which appears to trigger specific downstream effects. In the present study, we found that cGMP signal was initiated at 1 min, but that after 30 min the fluorescence intensity of cGMP was diffused in the cytoplasm. Mobilization of cGMP in the intracellular compartments suggests that it has a concerted regulatory role in pathophysiology.

In conclusion, our results demonstrate that, after ligand binding, NPRA is rapidly internalized and trafficked from the surface to the interior of cells and redistributed to early endosomes; then, traveling through lysosomal degradative pathways and a population of receptors, it is recycled back to the plasma membrane. The endocytic processes of NPRA and the mobilization of cGMP during receptor trafficking occur concurrently with the concerted regulatory action of this receptor to generate the intracellular signal. The eGFP-tagged receptor trafficking described in the present study should also be useful for studying these processes *in vivo.*

## Abbreviations

ANP: atrial natriuretic peptide
BNP: brain natriuretic peptide
CNP: C-type natriuretic peptide
DMEM: Dulbecco's modified Eagle's medium
EEA-1: early endosome antigen-1
eGFP: enhanced GFP
eGFP–NPRA: eGFP-tagged NPRA; GC-A/NPRA, guanylyl cyclase/natriuretic peptide receptor-A
HEK-293: human embryonic kidney-293
IB: immunoblotting
IBMX: 3-isobutyl-1-methylxanthin
IF: immunofluorescence
IFS: immunofluorescence staining
IP: immunoprecipitation
KHL: keyhole limpet haemocyanin
LAMP-1: lysosome-associated membrane protein-1
Rab11: a member of the Ras superfamily
Re: recycling endosome
